# Assessing context and readiness of emergency medicine physicians to promote evidence-based imaging referral guidelines: a mixed-methods study

**DOI:** 10.1093/tbm/ibaf035

**Published:** 2025-08-04

**Authors:** Yi Xiang Tay, Jeremy C P Wee, Marcus E H Ong, Shane J Foley, Robert Chun Chen, Lai Peng Chan, Ronan Killeen, Eu Jin Tan, May San Mak, Glenn Y H Ng, Yang Yann Foo, Jonathan P McNulty

**Affiliations:** Radiography and Diagnostic Imaging, School of Medicine, University College Dublin, Belfield, Dublin 4, Ireland; Radiography Department, Allied Health Division, Singapore General Hospital, Outram Road, Singapore 169608, Singapore; Division of Medicine, Department of Emergency Medicine, Singapore General Hospital, Outram Road, Singapore169608, Singapore; Duke-NUS Graduate Medical School, 8 College Road, Singapore 169857, Singapore; Division of Medicine, Department of Emergency Medicine, Singapore General Hospital, Outram Road, Singapore169608, Singapore; Duke-NUS Graduate Medical School, 8 College Road, Singapore 169857, Singapore; Radiography and Diagnostic Imaging, School of Medicine, University College Dublin, Belfield, Dublin 4, Ireland; Duke-NUS Graduate Medical School, 8 College Road, Singapore 169857, Singapore; Division of Radiological Sciences, Department of Neuroradiology, Singapore General Hospital, Outram Road, Singapore 169608, Singapore; National Neuroscience Institute, 11 Jalan Tan Tock Seng, Singapore 308433, Singapore; Duke-NUS Graduate Medical School, 8 College Road, Singapore 169857, Singapore; Division of Radiological Sciences, Department of Diagnostic Radiology, Singapore General Hospital, Outram Road, Singapore 169608, Singapore; St Vincent's University Hospital, Elm Park, Dublin 4, D04 T6F4, Ireland; School of Medicine, University College Dublin, Belfield, Dublin 4, Ireland; Duke-NUS Graduate Medical School, 8 College Road, Singapore 169857, Singapore; Division of Radiological Sciences, Department of Diagnostic Radiology, Singapore General Hospital, Outram Road, Singapore 169608, Singapore; Duke-NUS Graduate Medical School, 8 College Road, Singapore 169857, Singapore; Division of Radiological Sciences, Department of Diagnostic Radiology, Singapore General Hospital, Outram Road, Singapore 169608, Singapore; Division of Radiological Sciences, Department of Diagnostic Radiology, Singapore General Hospital, Outram Road, Singapore 169608, Singapore; Duke-NUS Graduate Medical School, 8 College Road, Singapore 169857, Singapore; Radiography and Diagnostic Imaging, School of Medicine, University College Dublin, Belfield, Dublin 4, Ireland

**Keywords:** implementation science, clinical decision rules, practice guidelines, emergency medicine, diagnostic imaging

## Abstract

**Background:**

Assessment of context and readiness to change are key components in the implementation of imaging referral guidelines.

**Purpose:**

In line with JBI’s (formerly known as the Joanna Briggs Institute) approach to evidence implementation, the aim of this study was to apply a mixed-methods study design to assess the context and readiness of physicians to use evidence-based imaging and referral guidelines, in tandem with associated opportunities and barriers.

**Methods:**

A survey was administered to physicians in an emergency department (ED) in Singapore, followed by virtual focus group sessions with physicians who volunteered. Mann–Whitney *U* test was used to evaluate differences in specialist and non-specialist responses. Braun and Clarke's reflexive thematic analysis was followed for data engagement, coding, and theme development.

**Results:**

Fourteen physicians responded to the survey, and 16 physicians participated in the focus groups. All physicians agreed that imaging utilization will increase in the coming decade, and most agree that overuse is a problem in the ED, especially conventional radiography (CR). Physicians gave a median score of 4 out of 5 to most questions evaluating their knowledge, skills, and attitude. There was no statistical difference in the scores between non-specialists and specialists, except for their preference for imaging guidelines that provide evidence to enhance clinical judgement (*P* = .03), where specialists had a higher mean rank. Key themes generated were workplace culture and factors influencing imaging referrals.

**Conclusion:**

Imaging overutilization in the ED, especially CR, is a problem. While physicians have the readiness, awareness, knowledge, skills, and attitude to change practice, factors such as workplace culture, medico-legal landscape, and interdisciplinary relationships impede such changes. The development of institutional guidelines coupled with targeted strategies and efforts involving key stakeholders is necessary to bridge the evidence-to-practice gap.

Implications

- **Practice:** Imaging referral guidelines, specifically institutional guidelines, can promote evidence-based imaging.
- **Policy:** Several factors, including the systems, staff, and choice of evidence-based intervention, drive the problem of imaging overuse in the ED.
- **Research:** Further research is needed to evaluate how various strategies can reduce the evidence-to-practice gap, thereby encouraging behavioural change in the use of imaging referral guidelines.

## Introduction

Diagnostic imaging plays a pivotal role in the comprehensive management of patients in an emergency department (ED), and several studies have demonstrated an upward trend in the utilization of diagnostic imaging in the ED, a pattern that may continue in the upcoming years [1–3]. Of concern is the increasing usage of ionizing radiation imaging modalities such as computed tomography (CT) and conventional radiography (CR) [1, 4]. While radiological investigations greatly contribute to improved patient care and outcomes, it is important to acknowledge that they can be costly and have adverse effects due to the use of ionizing radiation and contrast media [5, 6]. Simultaneously, they may also have limited value, as they neither enhance the diagnosis of pathologies nor increase hospital admissions [7, 8]. Furthermore, the utilization of low-value imaging or potentially inappropriate imaging results in increased unnecessary population radiation exposure, environmental costs, healthcare costs, incidental findings, and unnecessary interventions, which run counter to the objectives of value-based healthcare [9, 10].

The decision to proceed with imaging and the type of procedure requested are based on the individual clinician’s acumen and clinical impression. A study [11] conducted in the ED has revealed that there is unexplained and moderate variation in the utilization of imaging among emergency medicine (EM) physicians. This subject matter has been widely acknowledged as a complex issue, encompassing various intertwined issues and lacking a straightforward resolution [12]. Nevertheless, imaging decisions must be evidence-based and incorporate the best available research to facilitate informed decisions regarding the best imaging tests, thus ensuring appropriate and effective care for patients.

Evidence-based imaging referral guidelines have been developed by radiological (American College of Radiology Appropriateness Criteria, European Society of Radiology iGuide and The Royal College of Radiologists iRefer, etc) and non-radiological societies/bodies (Choosing Wisely, Image Wisely, etc) with other specialties globally as a strategic approach to promote value-based imaging, thereby enhancing healthcare quality and outcomes, while also exerting reducing healthcare costs [13]. Furthermore, it is known that timely access to appropriate imaging can affect the outcomes for a variety of medical conditions [14]. Implementing this strategy alongside other initiatives is crucial for promoting judicious utilization of imaging in the ED [12].

However, due to several impediments, such as competing policy priorities and entrenched cultural norms, physicians’ actual adoption of implementation knowledge may be limited, resulting in failure to translate evidence into routine clinical practice [15]. Implementation frameworks/models such as Reach, Efficacy, Adoption, Implementation, Maintenance (RE-AIM), Promoting Action on Research Implementation in Health Services (PARIHS), and JBI's (formerly known as the Joanna Briggs Institute) seven-phase process model can help to put evidence into practice [16]. Developed by an international, independent body of experts, JBI’s seven-phase process model [16] emphasized the importance of assessing context and readiness to change during the initial phase of an implementation project. It facilitates understanding of locally pertinent issues that are essential to practice change, identifies factors likely to influence the proposed change, and evaluates the organization's readiness to change [16]. Given this, the utilization of a mixed-methods research approach, which integrates qualitative and quantitative methodologies, is highly advantageous in facilitating an understanding and corroboration of the phenomenon [17].

In our case, there is a paucity of literature about EM physicians’ awareness, context, and readiness to change their use of imaging referral guidelines both internationally and locally, particularly within the institution. Therefore, the objective of this study was to apply a mixed-methods design to assess EM physicians’ context and readiness to change by first exploring their knowledge, skills, and attitudes towards evidence-based imaging and imaging referral guidelines to understand their readiness (i.e. Do physicians have the necessary knowledge and skills? Are they motivated to implement the change?). Subsequently, we further explored the opportunities and barriers to evidence-based imaging in the context of the ED. This will enhance our understanding of pertinent concerns within the local context that are crucial for practice change (implementation of imaging referral guidelines in the ED), while identifying factors that may affect the proposed change [16].

## Materials and Methods

Local approval to conduct the study was granted by the ED. The study was subsequently approved by University College Dublin, Human Research Ethics Committee (LS-CO-24-201-Tay-McNulty) as a low-risk study.

### Study design

The study was conducted from 1 June to 30 September 2024 and employed a mixed-methods research approach, incorporating both quantitative and qualitative components. The research utilized quantitative data obtained by a survey to establish a foundation for the research topic. Additionally, qualitative data collected through focus groups enables a deeper understanding of participants’ experiences and perspectives through group interaction.

### Study setting

The study was conducted in the ED of Singapore General Hospital, which is an institution affiliated with SingHealth Duke-NUS Academic Medical Centre in Singapore. On average, the ED receives more than 114,000 patients annually. This facility is a comprehensive 24-h ED that provides services for a wide range of emergencies, including major trauma. It offers direct access to laboratory services and diagnostic imaging, such as CT and CR.

The ED care team comprises EM specialists, supported by non-EM specialists such as residents (EM and non-EM), resident physicians, and medical officers. At the time of writing, there are a total of 34 EM specialists comprising Associate Consultants, Consultants, and Senior Consultants. Aside from the EM specialists, there are an average of 67 non-EM specialists (residents and some medical officers are on rotational posting) providing patient care.

### Recruitment

We disseminated the information sheet with the study objectives, methodologies, and invitation to EM physicians through meetings and emails. A convenience sample of EM physicians was recruited for the focus group (FG) sessions (Specialist and Non-specialist). Participation incentives (gift vouchers) were given to physicians who participated in the FG sessions.

### Survey data collection and data analysis

Survey items were developed through a review of radiology [18–23] and EM [24–27] literature, as well as through group discussions within the research team and with collaborators in the fields of radiology and EM (radiographers, experienced EM physicians, and radiologists). Prior to administration, the survey was piloted (to ensure reliability) and revised in terms of both its content and English wording (for clarity) based on feedback from peers in both departments. Furthermore, to enhance the validity of questions, particularly the subjective ones, we rephrased some questions and ensured the inclusion of several questions in various forms to evaluate the same subjective state.

The survey underwent three pilot runs, utilizing three to four participants during each iteration. We evaluated the comprehensibility and appropriateness of the survey items, ensuring that the questions were well-defined, well-understood, and consistently presented. Simultaneously, the participant information statements and consent forms presented on the initial page of the survey were assessed for comprehension. All comments were considered, any errors corrected, and the process reinstated until no other modifications were deemed required.

The survey employed a Likert scale for responses, along with multiple-choice and free-text formats. Seven questions in the survey gathered demographic information, while an additional four questions screened physicians’ perspectives on the over-utilization of imaging in the ED, knowledge of current imaging referral guidelines, and their opinion on the value of their individual imaging decisions. We posit that the evidence-based practice of applying imaging referral guidelines in clinical practice necessitates the acquisition and integration of knowledge, skills, and attitudes, as suggested by previous research [28]. Therefore, the remaining 30 questions on evidence-based imaging and imaging referral guidelines were grouped into knowledge, skills, and attitudes domains. These questions used 5-point Likert scale response choices ranging from “Strongly Disagree” to “Strongly Agree.”

We administered a web-based survey to EM physicians in the ED over a one-month duration. The survey was conducted using Qualtrics survey software (Qualtrics, Provo, USA) and consisted of an introductory section and consent form followed by the actual survey. For data analysis of the quantitative component, nominal variables were expressed using counts (percentages), while ordinal variables as medians (interquartile ranges [IQR]). The latter was compared using the Mann–Whitney *U* test, with a *P* < .05 taken as being statistically significant. Data were analysed with SPSS Statistics version 29.0 (IBM SPSS Inc., Chicago, USA).

### Focus group data collection and data analysis

To allow for a more nuanced and deeper exploration of the quantitative data collected, we also conducted five virtual FG sessions through Zoom (Zoom Video Communications Inc., San Jose, USA). FG was highly suitable in our current scenario as it encourages our participants to express their viewpoints on the study topic under discussion [29]. Furthermore, FG allowed for the strengthening and validation of preliminary findings from studies that used other research methodologies [29]. In order to address power differentials among potential participants, the FG participants were assigned to either a *Specialist* or *Non-Specialist* group based on their designation (homogenous group composition). This mitigated the impact of a power differential, which may otherwise discourage participants from speaking up during an FG due to concerns about the potential repercussions of expressing their views [29]. As no minimum sample size was recommended by Braun and Clarke, we established it pragmatically based on the research objectives and the available time and resources [30]. Nonetheless, we ensured an adequate sample size to achieve “information power” (the greater the information the sample holds, pertinent to the study, the fewer the number of participants needed) [31]. This was facilitated by our initial assessment of the study aim, sample specificity, use of established theory, quality of dialogue, and analysis strategy [31].

Since the FG involved convenience samples who volunteered (free from coercion) for participation, there were practical constraints such as difficulty in recruitment and the availability of physicians. Each FG consisted of two to four EM physicians, all working in the ED. A list of open-ended questions was prepared for the moderator to guide the discussion within the FG (see Supplementary Table S1). Similar to the survey questions, the topic guide was prepared through group discussions with colleagues in the field of radiology and EM (radiographers, experienced EM physicians, and radiologists). Other members of the research team reviewed the topic guide for clarity and comprehensiveness. As Y.X.T. (the first author) was someone unknown to the EM physicians, he took on the role of moderator to oversee and guide the discussion among participants, ensuring that ideas were shared and exchanged effectively. Additionally, the moderator also probed for further information and ensured that the conversation stayed focused and ensured the collection of rich data. Data generation was stopped based on the recommendations made by Braun and Clarke [32]. After assessing the data each participant provided and the variety of viewpoints collected, he determined that he had generated adequate data after five focus groups.

All FG sessions were audio-recorded with consent. We followed Braun and Clarke’s [33] reflexive thematic analysis (RTA) for data engagement, coding, and theme development. RTA emphasizes the significance of the investigator’s subjectivity as an analytical tool, as well as their reflexive involvement with theory, data, and interpretation; therefore, theme development is subjective but interpretive, making it well-suited for us to understand our participants’ experiences [33]. Our ontological stance was informed by realism, which meant that our participants’ accounts gave us direct access to how they experienced reality [34].

FG recordings were transcribed verbatim. Y.X.T.'s interpretation of the data was based on his perspectives as a radiographer with experience in value-based healthcare and implementation science. Y.X.T. read and re-read the transcript and wrote familiarization notes based on them [34]. He then coded the transcripts at both latent and semantic levels [34]. Candidate themes were constructed and revised and finalized based on Y.X.T. background, experience, and knowledge. This process was supported by Y.Y.F., an experienced RTA, non-healthcare professional researcher. Participants’ validation was not sought as ‘checking’ was not consistent with the RTA approach [35]. Similarly, the practices of data saturation and triangulation were also excluded [35]. Regarding the issue of qualitative quality criteria, we used Braun and Clarke’s Reflexive Thematic Analysis Reporting Guidelines (RTARG) [36].

## Results

### Quantitative results

Fourteen EM physicians participated in the survey, including nine non-specialists and five specialists. The majority of physicians (*n* = 11/14, 78.6%) were aged 30 years or older and predominantly received their undergraduate medical education in Singapore (*n* = 11/14, 78.6%) and practiced in a local ED (*n* = 11/14, 78.6%). They possessed 8.2 years (SD ± 6.3) of experience and spent 5.4 years (SD ± 3.9) in a Singapore ED. Table 1 illustrates the demographics of the survey respondents.

**Table 1.** Demographics

Designation	*n*/14	%
Medical officer	3	21.4
EM junior resident	3	21.4
Senior resident	3	21.4
Consultant	5	35.8
Age		
25–29 years old	3	21.4
30–34 years old	6	42.9
35–39 years old	4	28.6
50–54 years old	1	7.1
Country of undergraduate medical training		
Australia	1	7.1
Malaysia	1	7.1
Singapore	11	78.6
UK	1	7.1
Practiced in an overseas ED		
No	11	78.6
Yes	3	21.4
Speciality in EM		
No	3	21.4
Yes	11	78.6
Years of practice excluding housemanship (mean± SD)	8.2 years (± 6.3)	
Time spent in a Singapore ED in years (mean± SD)	5.4 years (± 3.9)	

All respondents anticipate an increase in the use of diagnostic imaging over the next decade, with the majority (*n* = 10/14, 71.4%) identifying overuse of imaging as a concern in the ED, with a substantial opportunity for improvement. Nonetheless, they self-reported that 71.21% (SD ± 14.96) of imaging referrals they made were high-value, with only two respondents (*n* = 2/14, 14.3%) indicating that they rarely use imaging referral guidelines. We observed a statistical difference (*U* = 7.50, *Z* =−2.23, *P* = .03) between non-specialists and specialists regarding their preference for “assistive” (providing evidence to enhance clinical judgement) imaging guidelines. The mean rank for the non-specialists was 5.83, while for specialists it was 10.5. Table 2 presents the results of the survey.

**Table 2.** Survey results

Screening questions			
Do you expect that diagnostic imaging utilization will increase in the coming 10 years?	*n*/14	%	
Yes	14	100	
Estimate the percentage of radiological investigations you refer that you feel are high-value: 0–100 scale? (mean± SD)	71.21% (± 14.96)		
Overutilization of imaging is a problem with a significant opportunity to improve in the ED	*n*/14	%	*P* value
Strongly disagree	1	7.1	.89
Somewhat disagree	1	7.1	
Neither agree nor disagree	2	14.3	
Somewhat agree	7	50.0	
Strongly agree	3	21.4	
Current usage, e.g. how frequently do you use evidence-based imaging guidelines to determine most appropriate radiological investigation?	*n*/14	%	*P* value
Rarely	2	14.3	.09
Sometimes	5	35.7	
Very often	7	50.0	

Questions on knowledge	Median [IQR]	*P*-value
I feel empowered when ordering radiological investigations	4.00 (3.00–4.00)	.10
I understand appropriate indications for diagnostic imaging	4.00 (4.00–5.00)	.81
I know the appropriate imaging technique choice that addresses the clinical question	4.00 (4.00–5.00)	.61
I have good understanding of the potential adverse effects of ionizing radiation	4.50 (4.00–5.00)	.09
I know the relevant clinical details to include in a radiological investigation	4.00 (4.00–5.00)	.35
I am able to suggest to patient an alternative (non-imaging, conservative management etc) to the imaging	4.00 (2.75–4.00)	.71
I know when to seek support/advice from a radiologist	4.00 (4.00–5.00)	.51
I am aware of the various evidence-based imaging guidelines	4.00 (4.00–4.25)	.80
I am able to apply the imaging guidelines when ordering a radiological investigation	4.00 (4.00–4.00)	.64
I have made radiological investigation(s) on behalf of doctors from other disciplines (specialist) in which I feel is not necessary	4.00 (3.75–5.00)	.35

Questions on skills	Median [IQR]	*P* value
I am confident about ordering radiological investigations for my patients	4.00 (4.00–5.00)	.88
I ensure that my radiological investigation request is appropriate and clinically accountable	4.50 (4.00–5.00)	.50
Where there are multiple imaging modalities for a specified clinical indication, I am confident that my selection is evidence-based	4.00 (3.00–4.00)	.14
I am confident about discussing my decision making for radiological investigation with my patients	4.00 (4.00–5.00)	.23
I am confident about discussing my decision making for radiological investigation with radiologists or radiographers	4.00 (4.00–4.25)	.22
I utilize evidence-based imaging guidelines as part of my routine practice	4.00 (3.75–4.25)	.10
I ensure that key clinical indications are included with every radiological investigation	4.50 (4.00–5.00)	.88
I prefer a “directive” (suggesting a course of action) imaging guidelines	4.00 (3.75–4.25)	.94
I prefer an “assistive” (providing evidence to enhance clinical judgement) imaging guidelines	4.00 (3.00–4.00)	**.03**
I depend on my own experiences rather than guidelines on how I approach imaging decisions	4.00 (3.00–4.00)	.47

Questions on attitudes	Median [IQR]	*P* value
I would like to be able to make sure that I am contributing to higher-value imaging by increasing the appropriateness of radiological investigations	5.00 (4.00–5.00)	.22
The use of clinical decision support based on evidence-based imaging guidelines will empower me and positively impact my clinical decision-making	4.50 (4.00–5.00)	.59
I am inclined to follow the recommendations from the imaging guidelines	4.00 (4.00–5.00)	.81
I weigh long-term risks against the immediate short-term potential diagnostic benefit	3.00 (2.00–4.00)	.41
Malpractice litigation is an important factor in my imaging decisions	4.00 (2.00–5.00)	.94
Patient satisfaction or patient insistence on imaging is an important factor in my imaging decisions	3.00 (2.00–4.00)	1.00
Before I order a radiological investigation, I would like to be provided with a ranking of the appropriateness of the imaging studies	4.00 (3.00–4.25)	.07
Application of imaging guidelines would improve patient outcomes	4.00 (4.00–5.00)	.38
Imaging guidelines would save time in imaging decision-making	4.00 (3.75–5.00)	.43
Imaging guidelines should be standalone	3.00 (2.00–3.25)	.40

5-point Likert scale: 1—Strongly Disagree, 2—Disagree, 3—Neither Agree nor Disagree, 4—Agree, 5—Strongly Agree.

### Qualitative results

Self-reported findings from our survey suggest that physicians possess proficient knowledge and skills in utilizing imaging referral guidelines and making imaging referrals; yet, the problem of inappropriate imaging persists. To probe further to understand the reason behind this phenomenon, we conducted five focus groups with various specialists (*n* = 9) and non-specialists (*n* = 7) EM physicians. Two salient themes were constructed: (1) Workplace culture seemingly favoured the use of diagnostic imaging, and (2) factors influencing the behaviour of physicians in making imaging referrals. Both themes also have sub-themes, with sub-themes in Theme 1 describing the various elements in the workplace culture and sub-themes in Theme 2 describing key factors that influenced physicians’ behaviour.

#### Theme 1: Workplace culture seemingly favoured the use of diagnostic imaging

Workplace culture encompasses shared beliefs and values instilled by leaders or influenced by everyone in the department. This theme describes the values and beliefs in the ED that influenced the referral decisions for diagnostic imaging for our participants.

1.1 Priorities in the ED

Many participants discussed how the healthcare sector, institutions, and downstream departments (such as specialist centres and allied health departments) were driven by inter-institutional competition to enhance patient experiences beyond conventional healthcare outcome measures. A few participants spoke about the shared ways of thinking about practicing frontloading in the ED for efficiency. Frontloading involves allocating a greater proportion of resources and/or effort at the beginning of the process and has been shown to improve patient wait time in the ED. Often, this entails referring patients for imaging procedures in the ED, either after triaging or following the initial consultation, regardless of its perceived value. By completing the diagnostic imaging at the ED, patients who will next consult specialists will not need to do it again. This saves them time, minimizes delays, and improves their experience (Table 3, Quote 1).

**Table 3.** Summary table of study themes with representative quotes

Theme 1: Workplace culture seemingly favoured the use of diagnostic imaging		
The values and beliefs in the ED that influenced the referral decisions for diagnostic imaging		
Sub-themes	Values and beliefs	Exemplar quotes
**Priorities in the ED**	Practicing frontloading in the ED can increase efficiency and patient experience	“This concept involves front-loading imaging for a patient. I believe that sometimes front loading may cause unnecessary imaging because the screening senior may not have done a thorough history and examination, which will be done more completely by the medical officer later on. However, the goal of front loading is to expedite the process, eliminating the need for the patient to wait twice. This allows for the completion of X-rays after triage, ensuring they are ready for the patient’s visit with the medical officer”. (Participant A)
**Priorities in the ED**	Diagnostic imaging are significant tools in current medical practice that supports safety and quality in healthcare	“I'm placing more orders because I've gained clinical experience over the years. And, of course, I have seen missed diagnoses. So that yeah. So perhaps I do order more CT imaging”. (Participant H)
Maximizing patient benefits	Diagnostic imaging optimizes patient benefits.	“Imaging expedites the diagnosis process, enables early patient pickup, and ensures prompt source control. Even for an unnecessary scan you perform, you might actually discover some pathology that you didn't previously detect”. (Participant A)
Maximizing patient benefits	Modern diagnostic imaging enhances patient care and preserves lives.	“Why does humankind advance? We have made advancements in technology and other areas. This is due to our desire to improve the quality of life for everyone. Why are we refusing something that could potentially benefit everyone?” (Participant E)
Safety of diagnostic imaging	Conventional radiography involves low ionizing radiation and is relatively safe and appropriate for most patients.	“So, for something that's very, very low radiation, you know X-rays; if he takes a plane, you get more cosmic radiation. Then I don't think it matters very much compared to”. (Participant I)

Theme 2: Factors influencing the behaviour of physicians in making imaging referrals		
Beyond workplace culture, there were key factors that influence imaging decision-making		
Sub-themes	Influence on decisions for diagnostic imaging	Exemplar quotes
Value of imaging referral guidelines	Imaging referral guidelines supported the imaging decision-making process and reduced fear of making an imaging referral.	“As a junior, I always believed that if there were clear guidelines in place, then I wouldn't be so afraid to ask for imaging”. (Participant H)
Prior experiences during practice	Personal experiences led to deviations in the practice of guidelines.	“We have seen cases with fractures, and we don't want to miss such types of cases where, based on guidelines, you didn't do the X-ray. Additionally, we often perform X-rays to rule out other pathologies, especially in the elderly population, as we don't want to overlook any practical issues”. (Participant L)
Limitation of imaging referral guidelines	Guidelines may not be inclusive or age-relevant, particularly for the elderly patient group who often have comorbidities and therefore clinical judgement supersedes in the imaging decision-making process.	“Why are we ordering it for so many people? A simple, low-cost chest X-ray could reveal a life-changing pathology. If you're experiencing MSK-like chest pain, it could be a sign of lung cancer. Unavoidable situations arise, such as when an elderly patient appears confused and giddy, only to discover that pneumonia is the cause of their nonspecific delirium. They may present with a slight giddiness, only to discover that they have a severe case of cannonball metastasis in their lungs, which is the cause of their symptoms”. (Participant A)
Limitation of imaging referral guidelines	In the current changing medico-legal climate, guidelines do not completely defend or absolve someone's liability, and therefore they offer imperfect protection to an individual using them.	“Then you also feel like all these complaints that you have to answer, even though you did the right thing by following the right rule, and the hospital will also support you. But even the answer to the complaint can be worth it; you ask yourself, is it worth it or not?” (Participant E)
Current medico-legal climate	Institutional-approved guidelines are deemed to be more relevant than international or professional body guidelines and could promote increased uptake and adherence, leading to a shift in physicians’ ordering behaviour while addressing their concerns about “protection.”	“Guidelines and workflows typically indicate approval at a higher level. So the people on the floor will also feel covered if they do not do something, and it's clearly stated that it's not necessary to do. You know you can order without fear, or rather not order without fear. And accepting a small risk of missing something where you know it will be a generally accepted behaviour. I think if you're a junior, you have that reassurance that you know you followed some sort of guideline, and there will be some support. Now, hopefully. That's how I think having institutional, approved guidelines with radiology backing will provide that sort of assurance”. (Participant H)
Sub-themes	Influence on decisions for diagnostic imaging	Exemplar quotes
Current medico-legal climate	Potential medical litigation or complaints from negative patient experience and care have contributed to the belief—err on the side of caution.	“I mean, misdiagnoses are very rare cases, but they exist. They do happen, so I think the issue is that because these have happened. We are more driven towards doing more investigations because there's really not much in doing more. And if you miss that one case, it becomes a problem for you. You probably think I should have just done it. It would have been so much easier”. (Participant O)
Consequence from non-compliance	Enforcement issues and lack of consequences for non-compliance behaviour.	“I guess it comes down to the consequences. If I fail to follow the guidelines, what's going to happen to me? If nothing is going to happen to me, then why should I follow the guidelines?” (Participant B)
Relationships with other stakeholders	The need to promulgate positive interdisciplinary relationships with other healthcare professionals	“I save the patient a small amount of radiation, the same amount he would receive if he were to walk in the sun or take off from a plane, but if I do so, I risk incurring the wrath of a colleague with whom I must continue working for a long time. It comes with a political cost. We want cordial relationships with the people we work with. So I will order imaging”. (Participant I)
Relationships with other stakeholders	Working collegially with other healthcare professionals for optimal transitions in care for patients.	“We had a trauma case, and we did a CT thorax and CTAP. After that, the orthopaedics requested a thoracic spine X-ray. I thought to myself, “But the CT scan already includes the thoracic spine.” You've got the entire spine down there. Why do you still need a plain film? Right? It doesn't make sense. And the reason was, if I don't order, my consultant will scold me, and we are trapped because it is after office hours. If I don't order an X-ray, the patient cannot proceed, although I think I did order in the end. But yeah. The pitiful cause originates from above, beyond the control of these subordinates. So, they have to follow. If my colleagues wish to streamline the patient journey, they should simply click, place an order, and complete the task. The patient exits the ED, marking the conclusion of the matter”. (Participant B)

Beyond satisfying the ED's demand for efficiency, more than half of the participants said their shared belief in current medical practice highlighted differences between their practice and evidence-based medicine (EBM). EBM involves the integration of the best available scientific evidence, the physician's expertise, and the patient's values to make optimal medical decisions that influence outcome measures, for instance, mortality, readmissions, effectiveness of care, and others. The deviation from EBM manifested in the request for imaging for patients who, according to evidence-based guidelines, did not warrant it. While conventional healthcare outcome measures, such as the timeliness of care, remained of paramount importance, there was a paradigm shift in medical practice in the current climate, where safety and quality are of increasing significance. Many participants described prior experiences, whether personal or observed, as contributing to culture in the ED, a field where certainty is elusive (Table 3, Quote 2).

1.2 Maximizing patient benefits

Diagnostic imaging was regarded as an essential investigation in a patient's journey within the ED and beyond. Consequently, requesting diagnostic imaging for patients was assumed to be a practice that promoted efficiency, safety, and timely care for patients. All participants spoke of the ways diagnostic imaging radically benefited the patients. The advantages for patients, such as expedited diagnosis, identification of life-changing pathology, accurate patient disposition, and enhanced patient reassurance, often underpinned the practice of performing diagnostic imaging in the ED. Consequently, the participants were predisposed to make imaging referrals to optimize patient benefits (Table 3, Quote 3).

Many participants also presumed that the use of modern diagnostic imaging technology was associated with safety and quality of patient care. They assumed that diagnostic imaging promotes safety and timeliness of care. Modern technology can provide physicians with clinical information unattainable by physical examination alone, enhancing patient diagnosis. For one participant, having imaging modalities such as CR and CT at disposal was a shift from the earlier days of medicine, where one relied on physical examination to make a diagnosis. This resonates with most participants, where several assert that contemporary imaging serves to enhance patient care and preserve lives. Moreover, imaging is now readily available in the ED; thus, physicians should use these tools effectively rather than rejecting the opportunity to enhance patient outcomes (Table 3, Quote 4).

1.3 Safety of diagnostic imaging

Physicians frequently requested imaging referrals for CR, which are readily available, low-cost, and involve low ionizing radiation. This visible manifestation in the ED was prominent, with more than half of the participants commenting on the high volume and prevalence of low-value imaging associated with CR. They also described the “protection” of paediatric patients and those of childbearing age, though the “protection” is often associated with CT, which involves a higher dose of ionizing radiation. Coupled with the aforementioned assumptions, participants frequently discounted the risk of low-ionizing radiation associated with CR by relating the radiation dose from these procedures similar to natural background radiation or plane travel. This led to the justification that CR imaging is relatively safe and appropriate for most patients, despite being low-value (Table 3, Quote 5).

#### Theme 2: factors influencing the behaviour of physicians in making imaging referrals

Understanding the workplace culture that seemingly favoured the use of diagnostic imaging did not mean that it defined the physician’s behaviour in making imaging referrals. What seemed essential was a sense that workplace culture guided the physicians in their decision to make imaging referrals both at the departmental and individual levels. However, there were key factors that also influenced their behaviour.

This theme focuses on the key factors that reportedly influence the participants’ imaging decision-making, complementing the quantitative data from the survey.

2.1 Value of imaging referral guidelines

Imaging referral guidelines from radiology professional bodies and other sources (e.g. Ottawa rules, Pittsburgh rules) have existed for some time; however, there is currently no local policy mandating the use of imaging referral guidelines in clinical practice. Despite this, more than half of the participants discussed their views on the benefits of these guidelines. They asserted that these structured guidelines aid in certain clinical decisions, helping them to be more objective in making imaging referrals. Additionally, it provides an avenue to justify and validate one’s imaging referrals while also supporting dialogue with patients on low-value imaging. A few junior participants expressed that the guidelines supported them in their imaging decision-making process, thereby reducing their fear of making an imaging referral (Table 3, Quote 6).

2.2 Prior experiences during practice

Many participants were not optimistic about their widespread use in clinical practice despite recognizing the benefits of imaging referral guidelines. They expressed suppositions that guidelines may not hold true in all clinical situations, necessitating deviation from the guidelines in practice. They attributed this behaviour to their personal experiences during clinical practice. Several participants discussed how their personal experiences led to deviations in the practice of guidelines (Table 3, Quote 7).

2.3 Limitation of imaging referral guidelines

There were also opinions on the validity of imaging referral guidelines for all different patient populations. Participants expressed concern that the guidelines may not be inclusive or age-relevant, particularly for the elderly patient group who often have comorbidities. This has led to the belief that guidelines are inadequate for this population group, primarily due to their generic nature and insufficient attention to issues specific to elderly patients with comorbidities. Therefore, participants often rely on clinical judgement in the imaging decision-making and treatment rather than applying guidelines (Table 3, Quote 8).

Participants were also hesitant to follow the recommendations from the imaging referral guidelines for another reason. As the number of lawsuits against medical practitioners rises, physicians are now grappling with a changing medico-legal climate that may cause worry. Many participants identified that the imaging referral guidelines did not adequately protect physicians during routine practice, and they expressed concern and a lack of confidence in the support and protection they would receive during legal defence. This stemmed from their belief that following guidelines does not completely defend or absolve someone's liability, and therefore it imperfectly protects the physicians (Table 3, Quote 9).

2.4 Current medico-legal climate

While imaging referral guidelines may be inadequate in certain cases and in terms of liability, physicians expressed strong support for institutional guidelines. A number of participants explicitly identify the development of institutional guidelines as a priority to encourage evidence-based imaging. This underscored the significance and applicability of institutional guidelines in comparison to international or professional body guidelines. Developed with various groups of stakeholders (transdisciplinary) who understood the local context, institutional-approved guidelines would be deemed as more relevant and could promote increased uptake and adherence, leading to a shift in physicians’ ordering behaviour while addressing their concerns about “protection” (Table 3, Quote 10).

Nonetheless, the growing number of complaints and cases that require physicians to appear before disciplinary tribunals due to misdiagnosis hinders any attempts at practice change, as participants, by their very nature, tend to stick to established practices and exercise caution. These deeper shared assumptions on potential medical litigation or complaints from negative patient experience and care have since contributed to their belief—err on the side of caution (Table 3, Quote 11).

2.5 Consequence from non-compliance

Some participants attributed their ongoing overutilization of imaging and non-compliance with guidelines to the lack of consequences (Table 3, Quote 12). As described previously, failure to diagnose a condition may result in punitive actions in the form of medio-legal implications; however, the current lack of audits and feedback reflects the lack of consequences for such behaviour and therefore steers participants toward overutilization of imaging, regardless of its perceived value. This spotlights enforcement issues and has implications for any forms of intervention introduced to resolve the problem.

2.6 Relationships with other stakeholders

Another factor that was well described was the need to maintain positive relationships and trust with other healthcare providers. Effective interdisciplinary relationships among healthcare teams are known to improve patient care and safety. However, such relationships can be difficult to maintain, and conflicts can arise between physicians and other healthcare providers. Some participants have described situations where imaging referral guidelines suggest that no imaging is necessary based on the clinical question and/or clinical examination; however, there were expectations from others to perform imaging or complete the referral process for a specific patient. The mismatch between the expectations of others and the physician's action when complying with guidelines had repercussions and could potentially impact relationships with other healthcare service providers. Consequently, to promulgate positive interdisciplinary relationships, participants chose to over-request imaging (Table 3, Quote 13).

In a similar vein, it was imperative that the well-being of patients take precedence above any personal conflict among healthcare professionals when deciding current and future treatment plans. ED physicians have to constantly engage and communicate with other specialists for patient disposition (destination of the patient within the care pathway after initial assessment/treatment), and thus working collegially with them is in their best interests. Suboptimal transitions in care can impact patients’ conditions, resulting in unnecessary burdens for both patients and the healthcare system. Therefore, some participants shared their experiences of attempting to resolve disputes with other healthcare providers, rather than insisting their position prevails at all costs (Table 3, Quote 14).

When reflecting on these key factors that influence their imaging decisions, physicians were described their interdisciplinary relationships, the medico-legal landscape, the limitations of imaging referral guidelines, and the carrot-and-stick approach. Alongside workplace cultures, these were crucial factors that must be addressed for physicians to change the utilization of imaging referral guidelines in practice. The full summary table of study themes with representative quotes can be found in Table 3.

## Discussion

The results of this study demonstrate that physicians self-report typically possessing proficient knowledge and skills in utilizing imaging referral guidelines and making imaging referrals in their practice. Furthermore, they exhibit encouraging attitudes towards the implementation of imaging referral guidelines and advocate value-based imaging. They highlighted factors that contribute to the excessive use of imaging, barriers to the application of imaging referral guidelines, and discussed strategies to promote value-based imaging.

The results from a recent survey involving radiologists and EM physicians on imaging overuse in the ED by Kwee *et al.* [18] resonate with our findings. Both studies identified factors influencing the overutilization of imaging in the ED, including malpractice litigation, patient pressure, inexperienced physicians, easy access/low barriers to imaging, and heavy patient load in the ED. Interestingly, Kwee *et al.*'s study did not describe the current practice of medicine, which we also identified through our thematic analysis as a common thread. Indeed, the belief that “certainty is unattainable in medicine” has a significant impact on the practice of ED physicians, the provision of standard care, and the propensity to “err on the side of caution.” Indeed, it was evident that physicians were uncomfortable with the absence of thorough investigation into all potential diagnoses and sought a definitive diagnosis for the patient. As a result, this has become a deeply ingrained culture, leading to an increase in the use of imaging.

Brandsæter *et al.* [37] performed a qualitative study examining stakeholders’ perspectives on the factors contributing to low-value imaging, highlighting patient expectations as an important driver. Although this aligns with our findings, ED physicians have also indicated that expectations from other healthcare providers, including general practitioners and specialists, also contribute to the prevalence of low-value imaging. While physicians are apprehensive about jeopardizing their professional standing and damaging their relationships with patients by declining to provide imaging referrals, they are also concerned about ruining their relationships with other service providers (physician-physician relationship) within the healthcare political landscape. Consequently, they were inclined to close the patient referral loop and build harmonious relationships with these providers by referring patients for imaging, whether for patient discharge, admissions, or follow-up. Undeniably, maintaining critical connections in these complex political arenas can pose challenges to value-based imaging [38].

Globally, CT is becoming more accessible and increasingly used in the ED, although the rates of increase differ [39]. Our study contributes to the literature by identifying the reasons for the increased use of CT imaging in the ED. Numerous physicians indicated that an accumulation of clinical experience over time has enhanced their confidence and competence in clinical examinations, thereby decreasing the reliance on CR for musculoskeletal injuries; however, this has concurrently led to an increased use of CT in the ED. Indeed, increased clinical experience and personal observations of missed diagnoses and unusual patient presentations have led to the use of CT imaging for a distinct patient profile. Moreover, as the final gatekeeper in the patient's journey, imaging may be necessary to ensure appropriate patient disposition and care. This was particularly significant in elderly patients and the semi-/nonurgent category, which Ha *et al.* [39] pointed out constitutes the highest proportion of ED visits. Many of these patients may potentially reside in the “grey” zone of imaging, contributing to the growth of imaging usage in the ED.

Many research studies have shown a deficiency in ionizing radiation awareness among ED physicians [20, 40, 41]. However, our results indicate that physicians self-reported a good understanding of the potential detrimental effects of ionizing radiation, although long-term risks were often not prioritized over the immediate short-term potential diagnostic benefits. This aligns with prior research indicating that physicians do not significantly prioritize radiation dose during imaging referral, when the imaging's influence on the patient's diagnosis, treatment, or future health is more critical [42]. Nonetheless, they effectively utilize their knowledge of ionizing radiation to engage patients in discussions that promote shared decision-making. Conversely, the principles of beneficence and non-maleficence in the practice of medicine may account for the less frequent ionizing radiation risk communication in situations where clinical needs outweigh the lifetime cancer risk. While there were more considerations in making imaging referrals for paediatric imaging and CT, most physicians deemed the impact of CR on lifetime cancer risk to be “negligible,” comparable to cosmic or background radiation. This contributed to the notion of a low threshold for making CR imaging referrals.

Despite other studies suggesting that CT accounts for the majority of imaging overuse in the ED [18, 43], our local physicians indicate that CR was the imaging modality with widespread prevalence of overuse. A local study on lumbar spine X-rays demonstrated a prevalence of overutilization, with 36.2% of imaging considered inappropriate [44]. The lower threshold for making imaging referrals, combined with the low levels of ionizing radiation, increased the utilization of these low-value imaging procedures. This contrasted with CT, which faced additional barriers in imaging referrals, including higher ionizing radiation, increased costs, and the local practice of involvement of senior physicians and radiologists as gatekeepers for imaging procedure approvals. Due to the scarcity of literature regarding the prevalence of low-value imaging in CR, additional investigations are necessary to fully understand the magnitude of the issue, which affects organizational policymakers, especially leaders in EM and radiology.

Imaging referral guidelines, specifically institutional guidelines, can promote evidence-based imaging; nevertheless, barriers and threats to its implementation must be addressed. We must avoid the traditional approach to implementation, frequently based on the “It Seemed Like A Good Idea At The Time” (ISLAGIATT) principle, which fails to recognize or address aspects essential for successful implementation. In this study, we assessed context and readiness to change, which is one of the phases in JBI’s seven-phase process model for evidence implementation [16]. Barriers and strategies identified by physicians will provide essential insights to assist healthcare policymakers in formulating efforts for change [45]. The current findings will be incorporated into the next phase of our research, an implementation project of imaging referral guidelines in the ED. Overall, there remains more to learn and explore about the implementation of imaging referral guidelines, with the aim of optimizing their usage to improve health outcomes. These findings may help address concerns about implementing imaging referral guidelines into practice. Further research is needed to evaluate how various strategies can reduce the evidence-to-practice gap, thereby encouraging behavioural change in the use of imaging referral guidelines.

### Strengths and limitations

Our study has several strengths, one of which is the use of qualitative techniques (FG) to improve our research design. Furthermore, the FG sessions were conducted according to best practice guidelines [29]. The moderator's role was assumed by an investigator (non-EM physician), ensuring that the discussion was not influenced by personal knowledge or experience and avoiding any power dynamics. While some may argue that the composition of the groups that participated in the FG sessions was not always representative of a larger or entire community, it is our belief that there was “representativeness” of the groups to the specific contexts and topic areas. We also followed recommendations for the number of FG sessions required for cross-group analysis to look for patterns and themes, as well as the number of participants in an optimum FG session [29]. Additionally, we iteratively analysed the FG data to revise the topic guide before moderating subsequent FGs. Moreover, the online survey provided a reliable means of inquiry as the questions presented to participants were standardized.

The small number of respondents for the survey and the involvement of a single institution may limit the generalizability of our study. Furthermore, the use of online surveys can potentially lead to skewed findings due to respondent bias, as we were unable to determine the motivations of those who participated [46]. Simultaneously, the homogeneous group composition for the FG sessions may result in potential “group think” or a lack of diversity in ideas, as well as hidden goals or power struggles inside a group [29]. Nevertheless, we presented our findings in a clear and objective manner, supported by evidence from the survey and focus groups.

## Conclusion

Imaging overuse in the ED (particularly CR overuse) is a problem according to most physicians and is driven by several factors, which include system, staff, and intervention. While physicians have the readiness, knowledge, skills, and attitude to use evidence-based imaging and imaging referral guidelines, workplace culture, medico-legal landscape, interdisciplinary relationships, and the lack of consequences may influence their imaging referral behaviour. Institutionally approved imaging referral guidelines can encourage changes in low-value imaging, but targeted strategies such as institutional guidelines, engagement, and carrot-and-stick approach are required to overcome these barriers and increase uptake. To empower our physicians to use imaging referral guidelines, a multitude of efforts involving all key stakeholders are required to bridge the evidence-to-practice gap.

## Supplementary Material

ibaf035_Supplementary_Data

## Data Availability

De-identified data from this study are not available in a public archive. De-identified data from this study will be made available (as allowable according to institutional IRB standards) by emailing the corresponding author.

## Welfare of Animals

This article does not contain any studies with animals performed by any of the authors.

## References

[1] Poyiadji N , Beauchamp N 3rd, Myers DT et al Diagnostic imaging utilization in the emergency department: recent trends in volume and radiology work relative value units. J Am Coll Radiol. 2023;20:1207–14. 10.1016/j.jacr.2023.06.033 37543154

[2] Christensen EW , Liu CM, Duszak R Jr, et al Association of state share of nonphysician practitioners with diagnostic imaging ordering among emergency department visits for medicare beneficiaries. JAMA Netw Open 2022;5:e2241297. 10.1001/jamanetworkopen.2022.41297 36355374 PMC9650604

[3] Juliusson G , Thorvaldsdottir B, Kristjansson JM et al Diagnostic imaging trends in the emergency department: an extensive single-center experience. Acta Radiol Open 2019;8:2058460119860404. 10.1177/2058460119860404 31392034 PMC6669846

[4] Maxwell S , Ha NT, Bulsara MK et al Increasing use of CT requested by emergency department physicians in tertiary hospitals in Western Australia 2003-2015: an analysis of linked administrative data. BMJ Open 2021;11:e043315. 10.1136/bmjopen-2020-043315 PMC7934721 33664075

[5] Stewart C , Smith-Bindman R. It is time to inform patients of medical imaging risks. JAMA Netw Open 2021;4:e2129681. 10.1001/jamanetworkopen.2021.29681 34643723

[6] Tay YX , Ong MEH, Foley SJ et al True cost estimation of common imaging procedures for cost-effectiveness analysis—insights from a Singapore hospital emergency department. Eur J Radiol Open 2024;13:100605. 10.1016/j.ejro.2024.100605 39498270 PMC11533038

[7] Rigney GH , King AH, Chung J et al Trends in non-focal neurological chief complaints and CT angiography utilization among adults in the emergency department. Intern Emerg Med 2024;19:2005–13. 10.1007/s11739-024-03569-9 38512433

[8] Pines JM. Trends in the rates of radiography use and important diagnoses in emergency department patients with abdominal pain. Med Care 2009;47:782–6. 10.1097/MLR.0b013e31819748e9 19536032

[9] Brady AP , Bello JA, Derchi LE et al Radiology in the era of value-based healthcare: a multi-society expert statement from the ACR, CAR, ESR, IS3R, RANZCR, and RSNA. Radiology 2021;298:486–91. 10.1148/radiol.2020209027 33346696

[10] Furlan L , Di Francesco P, Tobaldini E et al The environmental cost of unwarranted variation in the use of magnetic resonance imaging and computed tomography scans. Eur J Intern Med 2023;111:47–53. 10.1016/j.ejim.2023.01.016 36759306

[11] Valtchinov VI , Ip IK, Khorasani R et al Use of imaging in the emergency department: do individual physicians contribute to variation? AJR Am J Roentgenol 2019;213:637–43. 10.2214/AJR.18.21065 31063428

[12] Baloescu C. Diagnostic imaging in emergency medicine: how much is too much? Ann Emerg Med 2018;72:637–43. 10.1016/j.annemergmed.2018.06.034 30146444

[13] Tay YX , Foley S, Killeen R et al Impact and effect of imaging referral guidelines on patients and radiology services: a systematic review. Eur Radiol 2025;35:532–41. 10.1007/s00330-024-10938-7 39002059 PMC11632068

[14] Remedios D , Hierath M, Ashford N et al Imaging referral guidelines in Europe: now and in the future-EC Referral Guidelines Workshop Proceedings. Insights Imaging 2014;5:9–13. 10.1007/s13244-013-0299-8 24338616 PMC3948903

[15] The Lancet Global Health. Implementing implementation science in global health. Lancet Glob Health 2023;11:e1827. 10.1016/S2214-109X(23)00523-5 37973323

[16] Porritt K , McArthur A, Lockwood C et al JBI's approach to evidence implementation: a 7-phase process model to support and guide getting evidence into practice. JBI Evid Implement 2023;21:3–13. 10.1097/XEB.0000000000000361 36545902

[17] Johnson RB , Onwuegbuzie AJ, Turner LA. Toward a definition of mixed methods research. J Mixed Methods Res 2007;1:112–33. 10.1177/1558689806298224

[18] Kwee RM , Toxopeus R, Kwee TC. Imaging overuse in the emergency department: the view of radiologists and emergency physicians. Eur J Radiol 2024;176:111536. 10.1016/j.ejrad.2024.111536 38820950

[19] Kjelle E , Andersen ER, Brandsæter IØ et al Norwegian general practitioners’ and radiologists’ perspectives on the referral, justification, and unnecessary imaging-a survey. Scand J Prim Health Care 2024;42:574–81. 10.1080/02813432.2024.2366247 38916978 PMC11552244

[20] Hobbs JB , Goldstein N, Lind KE et al Physician knowledge of radiation exposure and risk in medical imaging. J Am Coll Radiol 2018;15:34–43. 10.1016/j.jacr.2017.08.034 29100884

[21] De Silva L , Baysari M, Keep M et al Patients’ requests for radiological imaging: a qualitative study on general practitioners’ perspectives. Health Expect 2023;26:2453–60. 10.1111/hex.13849 37587771 PMC10632629

[22] Borgen L , Stranden E. Radiation knowledge and perception of referral practice among radiologists and radiographers compared with referring clinicians. Insights Imaging 2014;5:635–40. 10.1007/s13244-014-0348-y 25164546 PMC4195837

[23] Bautista AB , Burgos A, Nickel BJ et al; American College of Radiology Appropriateness Do clinicians use the American College of Radiology Appropriateness criteria in the management of their patients? AJR Am J Roentgenol 2009;192:1581–5. 10.2214/AJR.08.1622 19457821

[24] Tung M , Sharma R, Hinson JS et al Factors associated with imaging overuse in the emergency department: a systematic review. Am J Emerg Med 2018;36:301–9. 10.1016/j.ajem.2017.10.049 29100783 PMC5815889

[25] Lumbreras B , Vilar J, Gonzalez-Alvarez I et al Evaluation of clinicians’ knowledge and practices regarding medical radiological exposure: findings from a mixed-methods investigation (survey and qualitative study). BMJ Open 2016;6:e012361. 10.1136/bmjopen-2016-012361 PMC5093629 27799242

[26] Griffey RT , Jeffe DB, Bailey T. Emergency physicians’ attitudes and preferences regarding computed tomography, radiation exposure, and imaging decision support. Acad Emerg Med 2014;21:768–77. 10.1111/acem.12410 25125272 PMC4135442

[27] Mills AM , Raja AS, Marin JR. Optimizing diagnostic imaging in the emergency department. Acad Emerg Med 2015;22:625–31. 10.1111/acem.12640 25731864 PMC4523049

[28] Baartman LKJ , de Bruijn E. Integrating knowledge, skills and attitudes: conceptualising learning processes towards vocational competence. Educ Res Rev 2011;6:125–34. 10.1016/j.edurev.2011.03.001

[29] Stalmeijer RE , McNaughton N, Van Mook WN. Using focus groups in medical education research: AMEE Guide No. 91. Med Teach 2014;36:923–39. 10.3109/0142159X.2014.917165 25072306

[30] Wutich A , Beresford M, Bernard HR. Sample sizes for 10 types of qualitative data analysis: an integrative review, empirical guidance, and next steps. Int J Qual Methods 2024;23: 10.1177/16094069241296206

[31] Malterud K , Siersma VD, Guassora AD. Sample size in qualitative interview studies: Guided by information power. Qual Health Res 2016;26:1753–60. 10.1177/1049732315617444 26613970

[32] Braun V , Clarke V. To saturate or not to saturate? Questioning data saturation as a useful concept for thematic analysis and sample-size rationales. Qual Res Sport Exerc Health 2021;13:201–16. 10.1080/2159676X.2019.1704846

[33] Braun V , Clarke V. One size fits all? What counts as quality practice in (reflexive) thematic analysis? Qual Res Psychol 2021;18:328–52. 10.1080/14780887.2020.1769238

[34] Terry G , Hayfield N, Clarke V, Braun V. Thematic analysis. In: Willig C, Rogers WS (eds), The SAGE Handbook of Qualitative Research in Psychology. London: SAGE Publications Ltd, 2017, 17–36.

[35] Braun V , Clarke V. Toward good practice in thematic analysis: Avoiding common problems and be(com)ing a knowing researcher. Int J Transgend Health 2023;24:1–6. 10.1080/26895269.2022.2129597 36713144 PMC9879167

[36] Braun V , Clarke V. Supporting best practice in reflexive thematic analysis reporting in palliative medicine: a review of published research and introduction to the Reflexive Thematic Analysis Reporting Guidelines (RTARG). Palliat Med 2024;38:608–16. 10.1177/02692163241234800 38469804 PMC11157981

[37] Brandsæter IØ , Andersen ER, Hofmann BM et al Drivers for low-value imaging: a qualitative study of stakeholders’ perspectives in Norway. BMC Health Serv Res 2023;23:295. 10.1186/s12913-023-09328-4 36978092 PMC10044073

[38] Waring J , Bishop S, Black G et al Understanding the political skills and behaviours for leading the implementation of health services change: a qualitative interview study. Int J Health Policy Manag 2022;11:2686–97. 10.34172/ijhpm.2022.6564 35297229 PMC9818121

[39] Ha NT , Abdullah L, Bulsara M et al The use of computed tomography in the management of injury in tertiary emergency departments in Western Australia: evidence of overtesting? Acad Emerg Med 2022;29:193–205. 10.1111/acem.14385 34480498

[40] Gunalp M , Gulunay B, Polat O et al Ionising radiation awareness among resident doctors, interns, and radiographers in a university hospital emergency department. Radiol Med 2014;119:440–7. 10.1007/s11547-013-0374-8 24356945

[41] Ditkofsky N , Shekhani HN, Cloutier M et al Ionizing radiation knowledge among emergency department providers. J Am Coll Radiol 2016;13:1044–9.e1. 10.1016/j.jacr.2016.03.011 27162040

[42] Borgen L , Stranden E, Espeland A. Clinicians’ justification of imaging: do radiation issues play a role? Insights Imaging 2010;1:193–200. 10.1007/s13244-010-0029-4 22347915 PMC3259314

[43] Dunne CL , Elzinga JL, Vorobeichik A et al A systematic review of interventions to reduce computed tomography usage in the emergency department. Ann Emerg Med 2022;80:548–60. 10.1016/j.annemergmed.2022.06.001 35927114

[44] Tay YX , Chan LL, Than SR et al Appropriateness of lumbar spine radiography and factors influencing imaging ordering patterns: paving the path toward value-driven health care. Int J Qual Health Care 2023;35: 10.1093/intqhc/mzad021 37043329

[45] Tay YX , Wee JCP, Ong MEH et al Optimising design of clinical decision support systems and implementation strategies to improve radiological imaging appropriateness—a qualitative study in the emergency department. Int J Med Inform 2025;202:105966. 10.1016/j.ijmedinf.2025.105966 40398269

[46] Andrade C. The limitations of online surveys. Indian J Psychol Med 2020;42:575–6. 10.1177/0253717620957496 33354086 PMC7735245

