# Supplementary material for: Assessing context and readiness of emergency medicine physicians to promote evidence-based imaging referral guidelines: a mixed-methods study
Source: Transl Behav Med. 2025 Aug 4;15(1):ibaf035. doi: 10.1093/tbm/ibaf035 (PMC12342136; doi:10.1093/tbm/ibaf035)
Supplement: ibaf035_Supplementary_Data [file ibaf035_supplementary_data.zip › FG Questions.docx]

Supplemental Material Table S1: List of Focus Group guiding questions

| **Focus Group Questions** | |
| --- | --- |
| 1. | Many in the survey felt that overutilisation of imaging is a problem with a significant opportunity to improve in the ED. Why might that be? |
| 2. | What are some of the strategies that you think may help to resolve this problem? How so? |
| 3. | In terms of patient satisfaction or patient insistence on imaging, how does it affect imaging decisions? |
| 4. | How about referrals made on behalf of doctors from other discipline (specialist)? What are the potential factors that may influence you making low-value/inappropriate radiological investigation(s) either for yourself or on behalf of doctors from other disciplines (specialist). |
| 5. | Studies have shown that among decision makers, novices function mostly in the analytic mode of reasoning, experiencing high levels of uncertainty and, therefore, account for the most variance. Do you feel the same? Why might that be? |
| 6. | When weighing the long-term risks of imaging (such as increased cancer risk from ionising radiation) against the immediate short-term potential diagnostic benefit, how does it influence your imaging decision? |
| 7. | In the survey, we asked you to estimate the percentage of radiological investigations you refer that you feel are high-value scale whereby high-value imaging refers to radiological investigations that impacts therapeutic decisions, improve patient out-comes and benefits for society as a whole. What are your thoughts on the variation of high-value imaging requested? Why might that be? |
| 8. | What are your beliefs around imaging guidelines for use in imaging decision? Tell me more about… |
| 9. | What do you see as the role of imaging guidelines? |
| 10. | What is your opinion on the potential value and effectiveness of evidence-based imaging guidelines? How might that be? |
| 11. | What are the potential barriers that prevented you from using imaging guidelines? |
| 12. | What can radiology do so that more and more clinicians are convinced by the imaging guidelines introduced from radiology societies? |
